# To Assess the Effect of Trauma on the Temporomandibular Joint in Postoperative Cases of Zygomaticomaxillary Complex Fractures

**DOI:** 10.1007/s12663-022-01826-y

**Published:** 2022-12-14

**Authors:** Mridula Sankaran, Chithra Aramanadka, Adarsh Kudva, Srikanth Gadicherla

**Affiliations:** 1 Oral and Maxillofacial Surgery, Dr.Priya’s Dental Studio, Kharghar, Mumbai, India; 2 Associate Professor, Department of Oral and Maxillofacial Surgery, Manipal College of Dental Sciences, MAHE, Manipal, Udupi, India; 3 International board certified, Associate professor, Department of Oral and Maxillofacial Surgery, Manipal College of Dental Sciences, MAHE, Manipal, Udupi, India; 4 Professor and Head, Department of Oral and Maxillofacial Surgery, Manipal College of Dental Sciences, MAHE, Manipal, Udupi, India

**Keywords:** Trauma, Temporomandibular joint, Postoperative, Zygomaticomaxillary complex, Mouth opening

## Abstract

**Aim and Objectives:**

The study aims to assess the incidence and features of temporomandibular joint(TMJ) dysfunction in post-surgical treatment of unilateral zygomaticomaxillary complex(ZMC) fractures.

The objectives are:

1. To assess severity of TMJ dysfunction in postoperative cases of ZMC fractures.
2. To create awareness of the same among clinicians.

**Methods:**

Patients presenting with zygomaticomaxillary complex fractures were evaluated prospectively. Evaluation of TMJ dysfunction was done by different parameters via questionnaire, clinical and radiographic examination preoperatively and a follow-up period of 1 week, 3 months and 6 months. The parameters were, clicking of joint, pain on opening /closing, pain on biting, deviation of mandible, pain in the preauricular region, ringing sound and mouth opening. Statistical analysis was done by the Friedman test and Post Hoc analysis.

**Results:**

On presentation, 69.1% patients diagnosed with ZMC fractures presented with symptoms related to TMJ dysfunction. Post-surgery 1 week majority findings persisted, with 21 patients complained of pain on opening or closing and 2 patients with a persistent opening click. These symptoms, however, decreased over the 3 month and 6 month follow up period. 5 patients presented with decreased mouth opening which was attributed to lack of adequate physiotherapy.

**Conclusion:**

Patients presented with mild symptoms of TMJ dysfunction until 6 months post-surgery, however these symptoms weren’t significant as the pain score assessed was found to decrease in the following post-operative periods. And the symptoms present were’nt exclusive to conclude a TMJ dysfunction. Early treatment and a close follow up are key to prevent progression of symptoms.

## Introduction

The frontal, sphenoid, temporal and maxillary bones articulate with the zygoma and provide strength and stability to the midface.

The forward projection of the zygoma causes it to be frequently injured and may be separated from its articulations resulting in a zygomaticomaxillary complex fracture. Aesthetic and functional deformities including persistent malar flattening, facial asymmetry and limited mandibular movement are indications for surgical treatment of zygomaticomaxillary complex fractures [1].

Complications following these fractures may be aesthetic, functional or neurosensory disturbances.

Difficulty in mouth opening commonly known as trismus perceived as temporomandibular dysfunction may be due to impingement of the displaced zygomatic body on the coronoid process or disturbance of the masticatory muscles resulting from trauma of the ZMC complex.

In general, the incidence rate of temporomandibular joint disorders after facial trauma is high and treatment timing plays a significant role in correction of the same [2]. Temporomandibular joint dysfunction as a result of zygomaticomaxillary complex fractures is less common than due to mandibular fractures.

Traumatic TMJ injuries left untreated may lead to complications like malocclusion, facial asymmetry and ankylosis. Difficulty in movements of the jaw including limitation in mouth opening and lateral excursive movements, deviation of the mandible and malocclusion manifest as complications of trauma related to the TMJ if left undiagnosed or untreated [3].

In this study, we have attempted to assess the symptoms of TMJ dysfunction post-surgery of ZMC fracture cases at different post-operative periods of follow-up.

## Materials and Methods

In this prospective study, 29 patients were included diagnosed with unilateral zygomaticomaxillary complex fractures and operated for the same by Open Reduction and Internal Fixation (ORIF) under general anaesthesia (GA) in the Dept of Oral and Maxillofacial Surgery, Manipal College of Dental Sciences, Manipal from June 2019 to October 2020. Patients with isolated unilateral ZMC fracture and who will be operated by ORIF were included in this study. Patients with bilateral ZMC fractures, history of TMJ disorders, conservatively managed fractures and other facial fractures were excluded.

1. *Preoperative Evaluation*: Thorough history taken from the patient including details of the trauma, associated comorbidities, history of loss of consciousness, vomiting, ear or nasal bleed and seizures. Clinical examination carried out in the form of general evaluation, preliminary and secondary evaluation of the ZMC including aesthetic features like facial asymmetry and functional features like malocclusion, pain on biting, diplopia and infraorbital step.
  Temporomandibular joint evaluation was based on clicking of the joint, pain score at the time of examination evaluated by the Visual Analogue scale, deviation on mouth opening, evaluation for symptoms pre-trauma and evidence of history of TMJ surgery performed.
2. *Radiographic Evaluation*: Patient’s radiographs were evaluated based on condylar position and texture.
3. *Surgical Procedure*: Keen’s approach and the lateral brow approach was used to expose the fracture. Reduction was done using a bone screw and Howarth’s elevator and fixation done using miniplates and self-tapping screws at the buttress and frontozygomatic suture.
4. *Postoperative Evaluation*: ZMC and TMJ evaluation were clinically and radiographically evaluated as preoperative assessment. However, emphasis was given on absence of depressed zygomatic body, absence of tenderness, step or mobility, decreased mouth opening post-surgery and any ear symptoms or muscle tenderness.
  Time of assessment:
  Pre-operatively
  One week post-operatively
  Three months post-operatively
  Six months post-operatively
5. *Questionnaire*: Patients were evaluated on the following questions-
  1. Is there a clicking sound on opening or closing the mouth?
6. Is there pain on opening or closing the mouth?
7. Is there pain on biting?
8. Is there deviation on opening the mouth?
9. Is there any pain associated to the pre-auricular region?
10. Is there any ringing sound in the ear?

All patients were recruited after ethical committee approval.

Informed consent was obtained from all patients before inclusion in the study

The collected data were further analysed by the post-hoc analysis -Wilcoxon test.

## Results

A total of 29 patients with ZMC fractures who presented to the Dept of Oral and Maxillofacial Surgery, Manipal from July 2019 to July 2020 were included in the study.

97% patients affected were males with no side predilection.

As per the criteria stated above the following results were obtained: (Table 1,2)

1. *Clicking sound*: Out of 29 patients evaluated, click sound was noted in 5 patients (17.2%) pre-operatively, of which 2 patients presented with click on opening. On the first week post-operatively, in 2 patients (6.9%) click sound was noted on opening. However, on the 3 months and 6 months follow-up, no click sound was noted in all patients.
2. *Pain on opening/closing the mouth*: Pre-operatively, 69% that is 20 out of 29 patients presented with pain on opening/closing the mouth. 1-week post-operatively, the statistics remained similar with 72.4%,3 months post-operatively, however, this parameter seemed to show a significant decrease with only 7 (24.1%) patients diagnosed with this finding. And this remained consistent, when 6 months post-operatively only 3.4% patients were found to have this finding.
3. *Deviation of the mandible*: wasn’t observed pre-operatively and remained consistent until 6 months post-operatively.
4. *Pain in relation to the preauricular region*: Following trauma, 37.9% patients presented with this finding, majority present in the same side as that of the fracture.1 week post-surgery, 41.4% patients presented with the above finding. However, 3 months and 6 months post operatively, this parameter was found to have a gradual decrease with a percentage of 10.3 and 3.4, respectively.
5. *Pain on biting*: As a sequelae of trauma, 82.8% patients presented with pain on biting pre-operatively.1 week post-surgery, due to surgical manipulation, a significant 37.9% patients presented with this feature. However, on 3-month and 6-month evaluation decrease in pain to almost nil was appreciated in all patients.
6. *Ringing sound in the ear*: none of the patients recruited in this study reported with this complaint pre-operatively, neither were found to have the same post-operatively at all 3 periods of follow-up.

**Table 1:** Depicting presence of different parameters pre-operatively and the following post-operative periods

Criteria	Response	Preop (%)	Post OP 1 (%)	Post OP 2 (%)	Post OP 3 (%)
Pain on openng/closing	Absent	31.0	27.6	75.9	96.6
	Present	69.0	72.4	24.1	3.4
Click sound	Absent	82.8	93.1	100.0	100.0
	Present	17.2	6.9	0.0	0.0
Deviation	Absent	100.0	100.0	100.0	100.0
	Present	0.0	0.0	0.0	0.0
Pain IRT preauricular region	Absent	62.1	58.6	89.7	96.6
	Present	37.9	41.4	10.3	3.4
Pain on biting	Absent	17.2	62.1	96.6	100.0
	Present	82.8	37.9	3.4	0.0
Ringing sound	Absent	100.0	100.0	100.0	100.0
	Present	0.0	0.0	0.0	0.0

**Table 2:** Depicting analysis of different parameters and P value determined by the Friedmann test

			Post hoc analysis—Wilcoxon test (p value adjusted)					
	Friedman test p value		POST OP 1–PREOP	POST OP 2–PREOP	POST OP 3–PREOP	POST OP 2–POST OP 1	POST OP 3–POST OP 1	POST OP 3–POST OP 2
Pain on opening/closing	0.000	HS	0.705	0.001	0.000	0.000	0.000	0.134
Click sound	0.008	HS	0.083	0.025	0.025	0.157	0.157	1.000
Deviation	1.000	NS	1.000	1.000	1.000	1.000	1.000	1.000
pain in relation to preauricular region	0.000	HS	0.655	0.005	0.002	0.003	0.001	0.157
Pain on biting	0.000	HS	0.000	0.000	0.000	0.012	0.001	0.317
ringing sound	1.000	NS	1.000	1.000	1.000	1.000	1.000	1.000

Pain score: on an average all patients rated a pain score of 4–5 pre-operatively (as depicted in the Table 3). 1 week post-operatively, an average pain score of 2.5 and an expected decrease of this value was noted in the following 3-month and 6-month follow-up period.

**Table 3:** Depiction of the mean of different parameters and comparison of P value by post-Hoc analysis and the Wilcoxon test

	N	Mean	Std. Deviation	Median (IQR)	Friedman test p value	Post Hoc* analysis—Wilcoxon test (p value adjusted for Bonferroni correction)*					
						Pre–Post op 1	Pre–Post op 2	Pre–Post op 3	Post op 1–2	Post op 1–3	Post op 2–3
Pain –PREOP	29	4.07	1.85	4(2.5–6)	0.000, HS	0.000, HS	0.000, HS	0.000, HS	0.000, HS	0.000, HS	0.058, NS
Pain–POST OP 1	29	2.28	1.10	2(1–3)							
Pain–POST OP 2	29	0.52	0.74	0(0–1)							
Pain–POST OP 3	29	0.31	1.14	0(0–0)							

Condylar texture was also assessed pre-operatively to rule out any pre-existing TMJ pathology and to analyse the effect of trauma directly on the TM joint in the form condylar erosion until one week post-operatively.

However, this finding was found to be non-significant in the follow-up period.

ZMC Evaluation:

On extraoral examination, in almost 40% cases the patient’s complaint was a depressed malar.

During the 3-month and 6-month follow-up period, 1 patient reported back with a persistent aesthetic finding of a depressed malar region. This was, however, owed to the comminuted nature of fractures in this patient.

Mouth opening and mandibular movements, diplopia, restriction in gazes, subconjunctival haemorrhage, healing of extraoral lacerations/ incisions if any, and presence of any obvious contour defect was noted.

Mouth opening:

an average of 63% patients presented with difficulty in mouth opening pre-operatively which was owed to a variety of factors.

One of the main goals of surgery was to improve the mouth opening.

On follow-up of 1 week, improvement in mouth opening was seen in all patients.

However, on the 3 month and 6 month review,20% patients presented with a decrease in this parameter, which was found to be due to inadequate physiotherapy by the patient.

## Discussion

High incidence of fractures of the zygoma is due to the tetrapod configuration and the various buttresses making it prone to trauma owing to a variety of causes including road traffic accidents, assault, accidental falls, sports injuries etc. [4]

Trismus or difficulty in mouth opening is frequently attributed to the spasm of masticatory muscles which is included in the extrinsic causes for TMJ dysfunction post trauma [5].

Symptoms of TMJ disorders generally present in the form of tenderness in relation to pre-auricular region, opening/ closing click, recurrent TMJ dislocation, restricted mouth opening and orofacial pain [6].

In this study, on history taking none of the patients presented with a previous TMJ surgery or any of the symptoms as mentioned above.

Following surgery of ZMC fractures, clinical signs of dysfunction were seen only in 30% patients till 3 months post-operatively, however, 2 patients showed similar symptoms till 6 months post-surgery.

These findings differed to the study by Rajantie et al. [7], according to which 38 out of 45 patients were found to have TMJ dysfunction symptoms until the 6 month follow-up period. The reason for which was found to be a difference in the variables examined, mechanism of injury and severity of the fracture. [6]

In studies by Riberio et al., the strength of the masticatory muscles was evaluated post-surgery of zygomatico-orbital complex fractures in which it was found that the muscles of the affected side reached their 70% capacity as compared to the opposite side with the analysis of bite force, electromyography and mandibular mobility. [8]

Pain score assessment conducted by the visual analogue scale in our study was found to decrease post-operatively significantly and hence the pain on opening and closing and in relation to the preauricular region was found to be present but mild as compared with previous assessment.

Hence our findings differed from that of the study by Rajantie et al. who found significant TMJ dysfunction until 6 months follow-up.

A study by Charles E. Anyanechi showed that the occurrence of temporomandibular joint disorders was higher in patients diagnosed and treated for isolated zygomatic complex and isolated mandibular fractures.

The factors playing an essential role in the incidence of temporomandibular dysfunction are treatment timing and the method of treatment. Improper reduction and/or immobilization leads to complications like malunion which in turn affects muscle activity and hampers joint movement [9].

Stelea et al. outlined a treatment plan dividing it into 2 broad categories-surgical and non-surgical treatment. Non-invasive therapy (pharmacotherapy, physical therapy) can be employed and coupled with minimally invasive methods for the effective recovery of post-traumatic TMJ disorders [10].

As proposed by Chang C-M, et al., adequate preoperative imaging including the TMJ area, thorough history and examination, regular postoperative physiotherapy follow-up and observation of mouth opening improvement would serve as a protocol in preventing the risk of complications in ZMC fractures involving the TMJ [11].

There were also certain limitations to our study including some patients were lost during follow-up due to the present COVID-19 crisis, no particular index was used for evaluation and masticatory muscle function was not assessed as a separate parameter.

40 patients met with the inclusion criteria, however, only 29 patients complied with follow-up protocol. Further studies need to be conducted for thorough evaluation, diagnosis and appropriate management of the same.

## Conclusion

A significantly smaller number of patients operated for ZMC fractures in this study showed TMJ dysfunction symptoms until 6 months follow-up and the symptoms presented as described above is not suggestive of a sign of TMJ dysfunction in particular and may be due to other factors including injury to the soft tissues.

Close follow-up of such patients will help diagnose and manage the symptoms more promptly and accurately. Further studies need to be conducted for a more detailed evaluation of TMJ dysfunction, which will help provide treatment at early stages.
